# Supplementary material for: Ribosome heterogeneity arising from common and rare rRNA sequence variants affects diverse human phenotypes
Source: medRxiv. 2025 Nov 19:2025.09.02.25334953. Originally published 2025 Sep 4. Preprint. [Version 2] doi: 10.1101/2025.09.02.25334953 (PMC12424869; doi:10.1101/2025.09.02.25334953)
Supplement: 1 [file NIHPP2025.09.02.25334953V2-supplement-1.pdf]

medRxiv preprint doi: https://doi.org/10.1101/2025.09.02.25334953; this version posted November 19, 2025. The copyright holder for this preprint (which was not certified by peer review) is the author/funder, who has granted medRxiv a license to display the preprint in perpetuity.

It is made available under a CC-BY 4.0 International license .

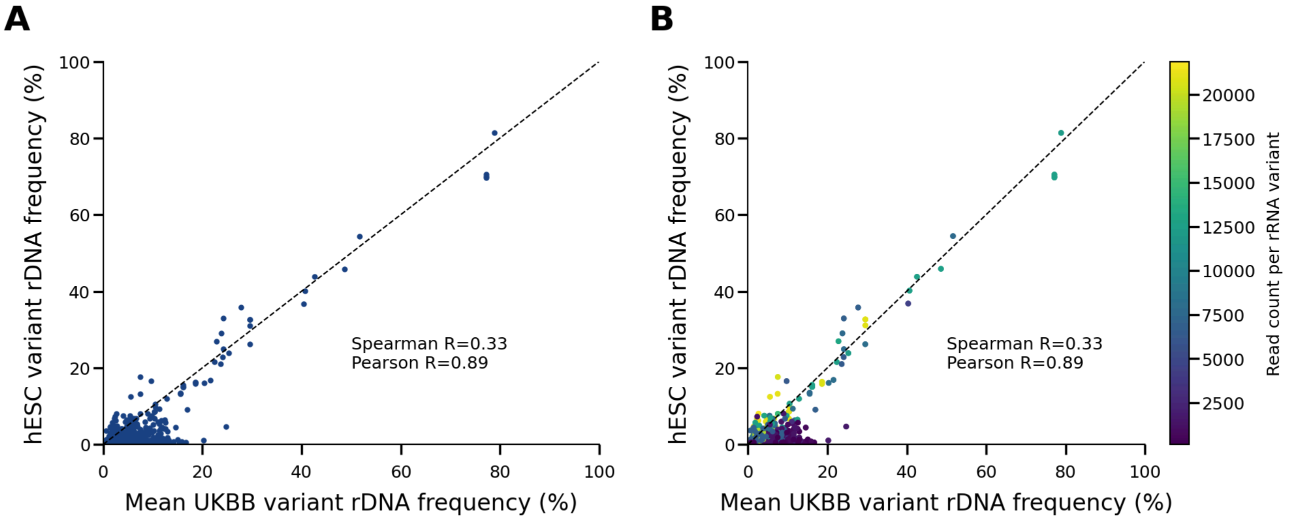

Figure S1: rRNA variants frequencies in hESC cell line and average UKBB profile agree for high coverage sequencing variants

(A) Scatter plot comparing the genomic rRNA variant profile of two datasets: mean UKBB and the hESC cell-line. Spearman and Pearson correlations are presented.

(B) Similar to (A) but variant frequencies are colored by the number of reads that were mapped to the rRNA variant. A color bar indicating read count is presented.

medRxiv preprint doi: https://doi.org/10.1101/2025.09.02.25334953; this version posted November 19, 2025. The copyright holder for this preprint (which was not certified by peer review) is the author/funder, who has granted medRxiv a license to display the preprint in perpetuity.

It is made available under a CC-BY 4.0 International license .

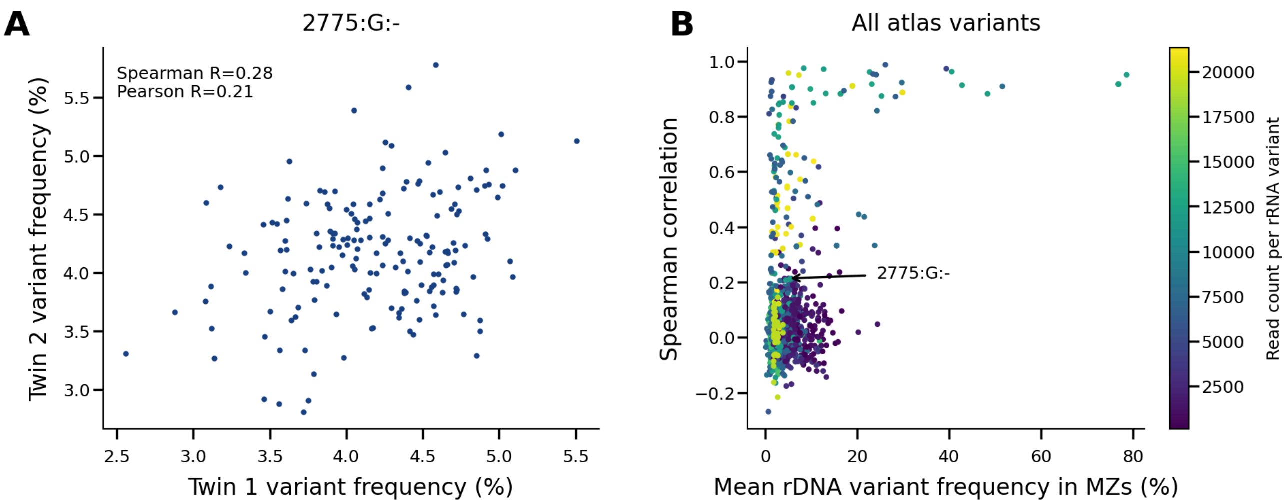

Figure S2: rRNA variants contain somatic sequence variations

(A) Scatter plot showing the correlation of variant frequencies between monozygotic twins for the variant 2775:C:T of the 28S gene. The Spearman correlation coefficient (R = 0.28) and Pearson correlation coefficient are indicated, demonstrating low heritability for this specific variant.

(B) Scatter plot showing the Spearman correlations of all atlas variants against their mean rDNA variant frequency in monozygotic twins. The Spearman correlation for variant frequencies are colored by the number of reads that were mapped to the rRNA variant. A color bar indicating the mean read count per sample is presented. The variant 2775:C:T from panel (A) highlighted.

medRxiv preprint doi: https://doi.org/10.1101/2025.09.02.25334953; this version posted November 19, 2025. The copyright holder for this preprint (which was not certified by peer review) is the author/funder, who has granted medRxiv a license to display the preprint in perpetuity.

It is made available under a CC-BY 4.0 International license .

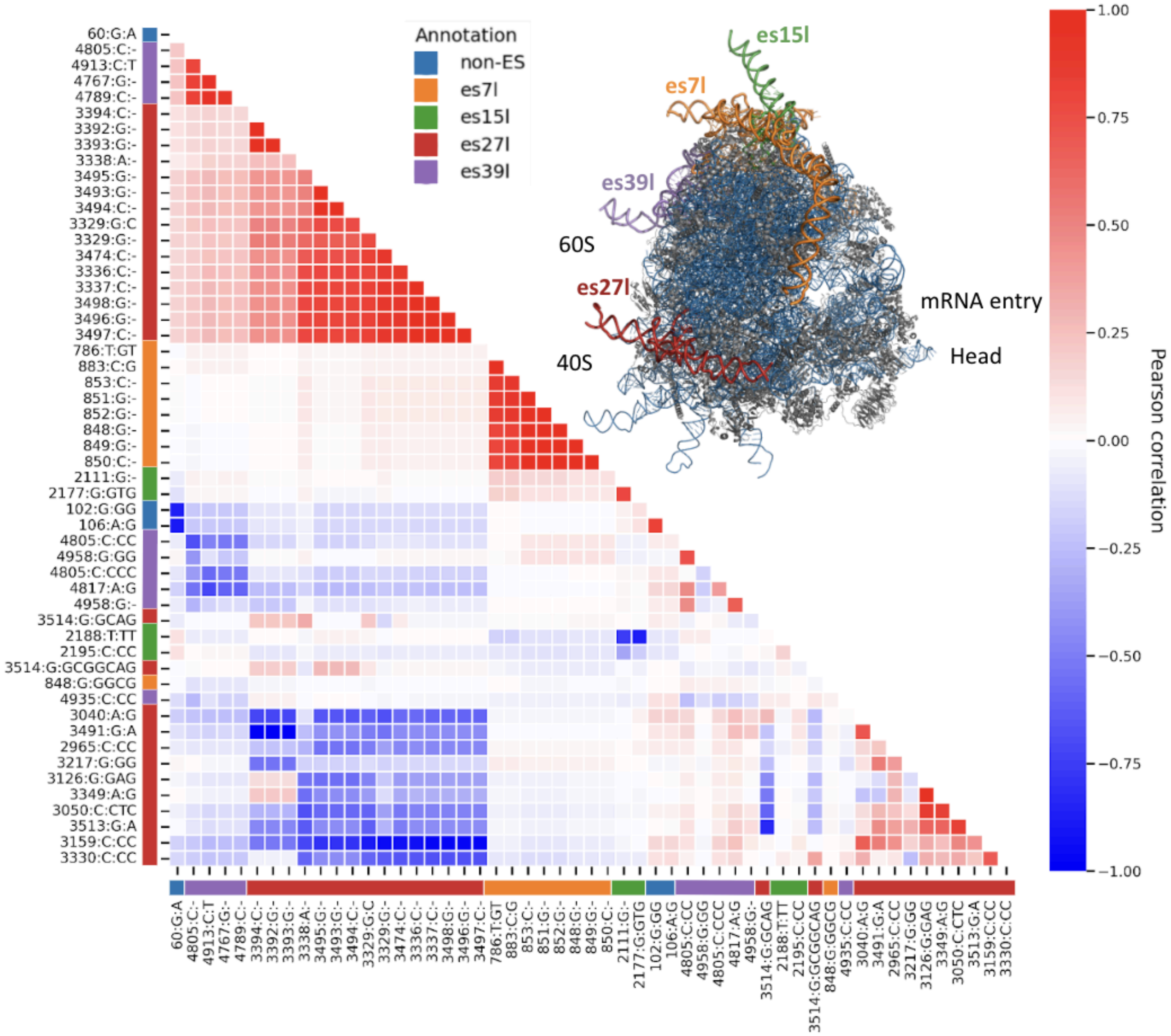

Figure S3: rRNA variants shows some haplotype linkage between rRNA regions
Heatmap displaying high heritable variants correlation to one another after hierarchical clustering. The color gradient represents the Pearson correlation values. A structure of a ribosome (PDB 4v6x [27]) with ES/non-ES region indicating the specific expansion segments (es7l, es15l, es27l, es39l) and non-ES variants in different colors. These colored annotations or ribosome regions are also annotated in the rows and columns of the heatmap.

medRxiv preprint doi: https://doi.org/10.1101/2025.09.02.25334953; this version posted November 19, 2025. The copyright holder for this preprint (which was not certified by peer review) is the author/funder, who has granted medRxiv a license to display the preprint in perpetuity.

It is made available under a CC-BY 4.0 International license .

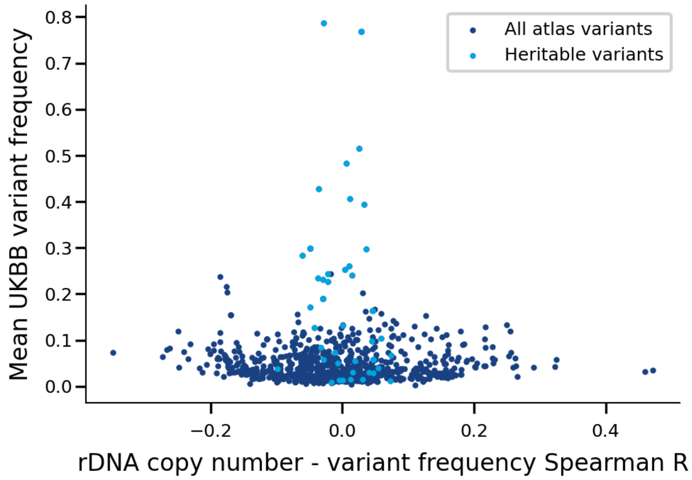

Figure S4: Heritable rRNA variants and rDNA copy numbers are not well correlated
Scatter plot displaying the Spearman correlation between rDNA copy number estimates and rRNA variant frequencies on the X-axis, and the mean UKBB rRNA variant frequency on the Y-axis.

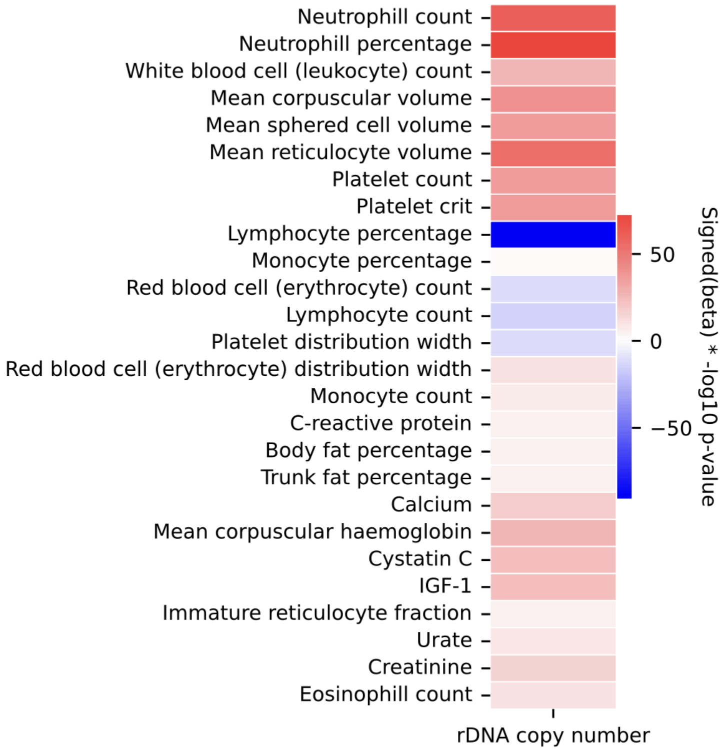

Figure S5: rDNA copy numbers associate with blood related traits
Heatmap of p-values for the association of rDNA copy numbers with blood traits. The heatmap color gradients indicate significance levels for each trait. The colors red and blue indicate the sign of the linear regression beta where red indicates positive association with the trait and blue for negative association.

medRxiv preprint doi: https://doi.org/10.1101/2025.09.02.25334953; this version posted November 19, 2025. The copyright holder for this preprint (which was not certified by peer review) is the author/funder, who has granted medRxiv a license to display the preprint in perpetuity.

It is made available under a CC-BY 4.0 International license .

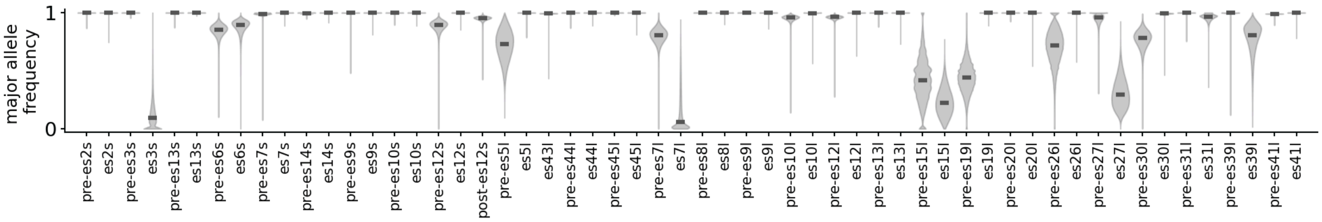

Figure S6: Major allele frequency of ES and non-ES regions of the 18S and 28S.
Distribution of the frequencies of the most abundant allele (major allele frequency; MAF) within the UK Biobank cohort. Segments are ordered by their positions in the 18S and 28S (Table S4).
