# Supplementary material for: rRNA paralogs with variations, rRNA-subtypes, affect diverse human phenotypes
Source: medRxiv. 2025 Sep 4:2025.09.02.25334953. Preprint. [Version 1] doi: 10.1101/2025.09.02.25334953 (PMC12424869; doi:10.1101/2025.09.02.25334953)
Supplement: Supplementary file 1 [file NIHPP2025.09.02.25334953V1-supplement-1.pdf]

medRxiv preprint doi: https://doi.org/10.1101/2025.09.02.25334953; this version posted September 4, 2025. The copyright holder for this preprint (which was not certified by peer review) is the author/funder, who has granted medRxiv a license to display the preprint in perpetuity.
It is made available under a CC-BY 4.0 International license .

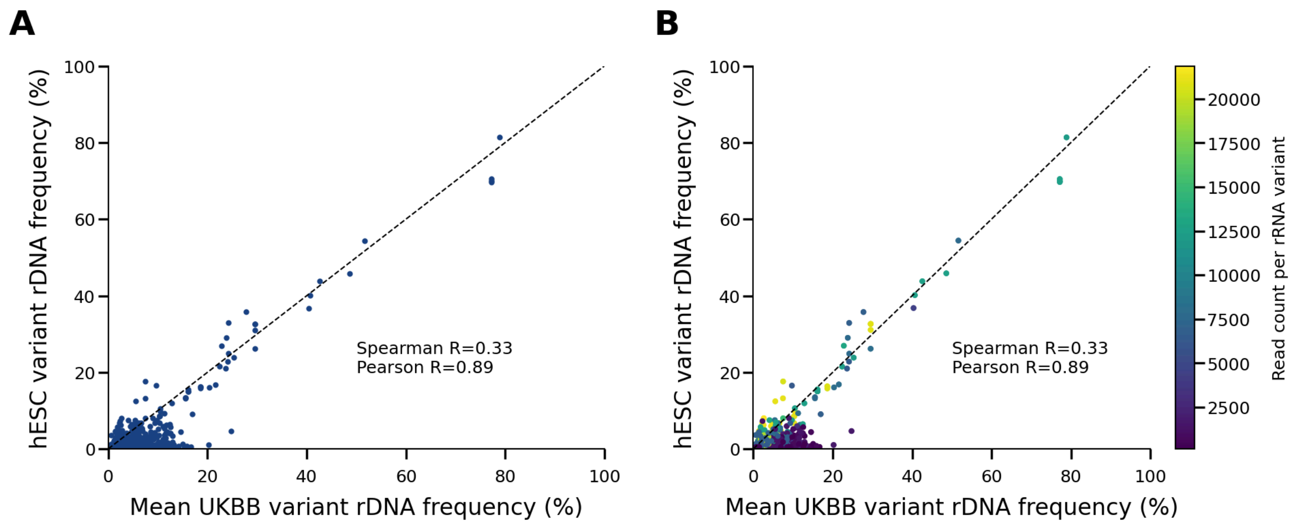


**Figure S1: rRNA variants frequencies in hESC cell line and average UKBB profile agree for high coverage sequencing variants**

(A) Scatter plot comparing the genomic rRNA variant profile of two datasets: mean UKBB and the hESC cell-line. Spearman and Pearson correlations are presented.

(B) Similar to (A) but variant frequencies are colored by the number of reads that were mapped to the rRNA variant. A color bar indicating read count is presented.

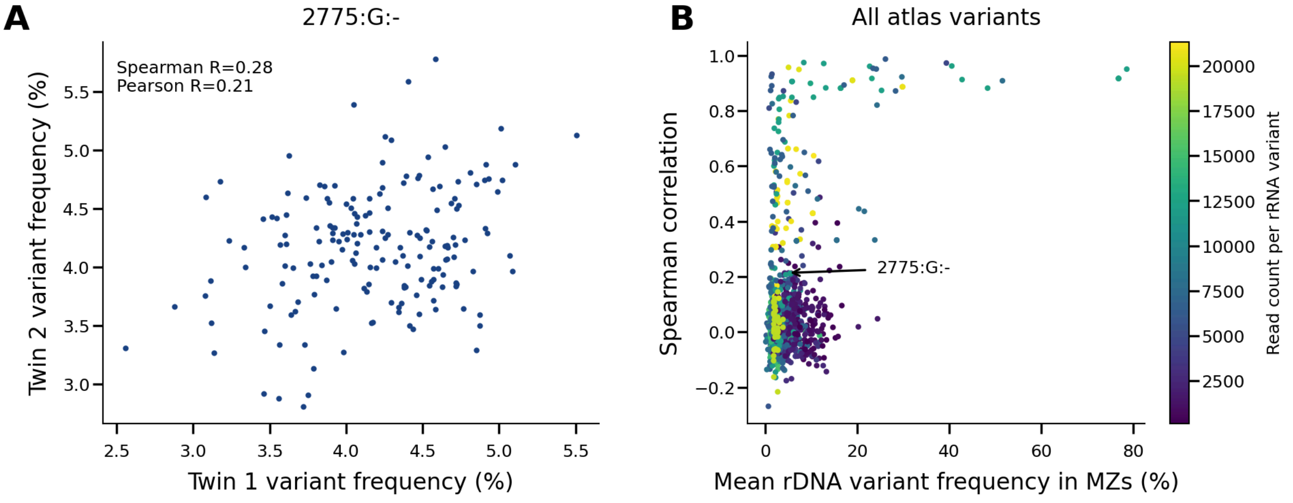

medRxiv preprint doi: https://doi.org/10.1101/2025.09.02.25334953; this version posted September 4, 2025. The copyright holder for this preprint (which was not certified by peer review) is the author/funder, who has granted medRxiv a license to display the preprint in perpetuity.
It is made available under a CC-BY 4.0 International license .

**Figure S2: rRNA variants contain somatic sequence variations**

(A) Scatter plot showing the correlation of variant frequencies between monozygotic twins for the variant 2775:C:T of the 28S gene. The Spearman correlation coefficient (R = 0.28) and Pearson correlation coefficient are indicated, demonstrating low heritability for this specific variant.

(B) Scatter plot showing the Spearman correlations of all atlas variants against their mean rDNA variant frequency in monozygotic twins. The Spearman correlation for variant frequencies are colored by the number of reads that were mapped to the rRNA variant. A color bar indicating the mean read count per sample is presented. The variant 2775:C:T from panel (A) highlighted.

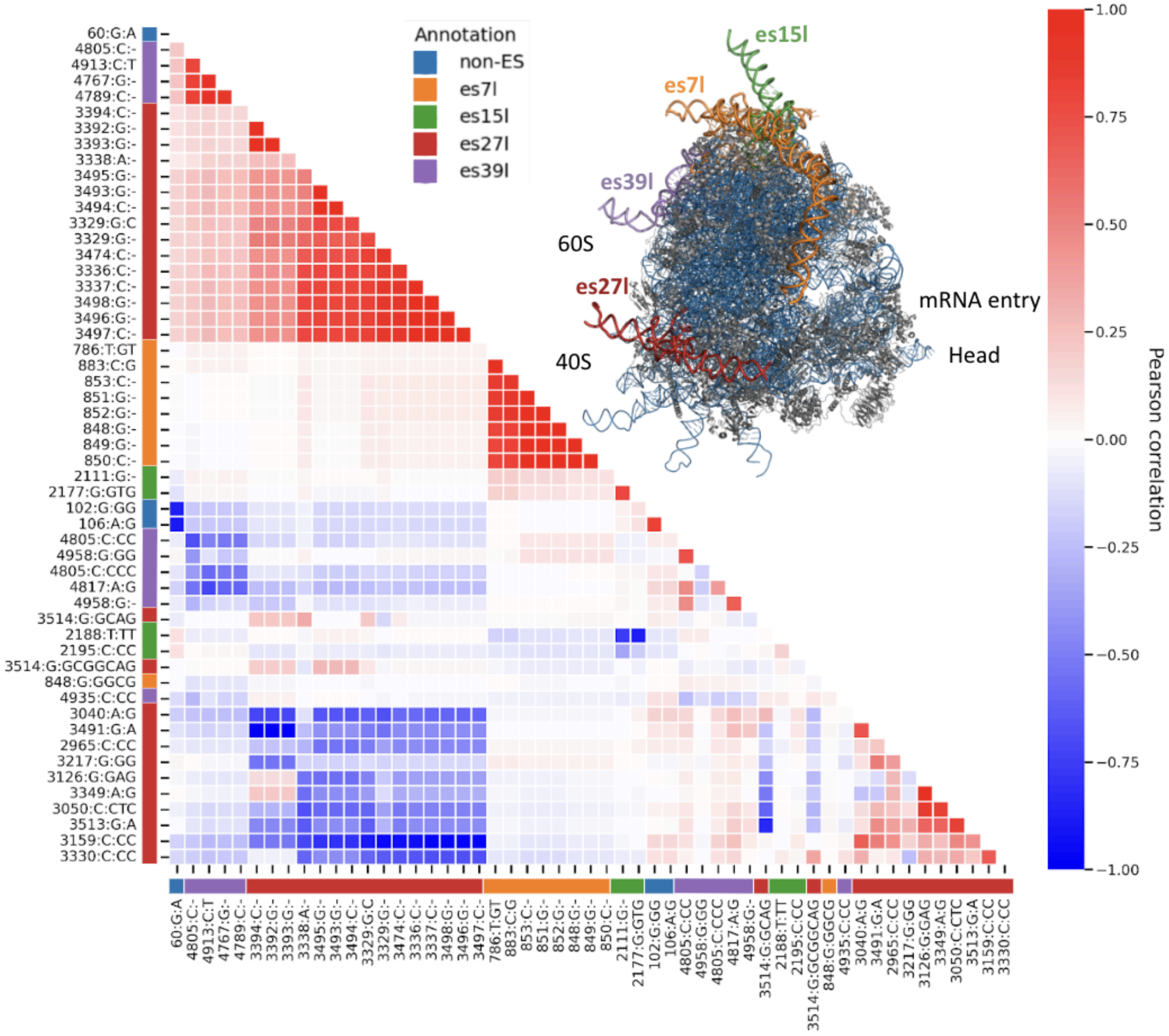


**Figure S3: rRNA variants shows some haplotype linkage between rRNA regions**

Heatmap displaying high heritable variants correlation to one another after hierarchical clustering. The color gradient represents the Pearson correlation values. A structure of a ribosome (PDB 4v6x [27]) with ES/non-ES region indicating the specific expansion segments (es7l, es15l, es27l, es39l) and non-ES variants in different colors. These colored annotations or ribosome regions are also annotated in the rows and columns of the heatmap.
